# Screening of Peptide Ligands for Pyrroloquinoline Quinone Glucose Dehydrogenase Using Antagonistic Template-Based Biopanning

**DOI:** 10.3390/ijms141223244

**Published:** 2013-11-25

**Authors:** Koichi Abe, Wataru Yoshida, Kotaro Terada, Yukiko Yagi-Ishii, Stefano Ferri, Kazunori Ikebukuro, Koji Sode

**Affiliations:** Department of Biotechnology, Graduate School of Engineering, Tokyo University of Agriculture & Technology, 2-24-16 Naka-cho, Koganei, Tokyo 184-8588, Japan; E-Mails: abekou@cc.tuat.ac.jp (K.A.); wyoshida@cc.tuat.ac.jp (W.Y.); kterada88@hotmail.com (K.T.); yagimay@hotmail.com (Y.Y.-I.); stefano@cc.tuat.ac.jp (S.F.); ikebu@cc.tuat.ac.jp (K.I.)

**Keywords:** peptide ligand, phage display, site-directed, inhibitor

## Abstract

We have developed a novel method, antagonistic template-based biopanning, for screening peptide ligands specifically recognizing local tertiary protein structures. We chose water-soluble pyrroloquinoline quinone (PQQ) glucose dehydrogenase (GDH-B) as a model enzyme for this screening. Two GDH-B mutants were constructed as antagonistic templates; these have some point mutations to induce disruption of local tertiary structures within the loop regions that are located at near glucose-binding pocket. Using phage display, we selected 12-mer peptides that specifically bound to wild-type GDH-B but not to the antagonistic templates. Consequently, a peptide ligand showing inhibitory activity against GDH-B was obtained. These results demonstrate that the antagonistic template-based biopanning is useful for screening peptide ligands recognizing the specific local tertiary structure of proteins.

## 1. Introduction

Phage display is well known as a powerful tool for screening peptides or proteins that bind to various targets [1–4], such as proteins [5], DNA [6], biomaterials [7], or inorganic materials [8,9]. Especially, peptides can be easily screened by commercialized phage display library kits. Peptide ligands selected by phage display have not only been applied as protein inhibitors [10,11] and molecular recognition elements for biosensors [12,13], but also for the improvement of protein function, such as enzymatic activity [14], stability [15], or substrate specificity [16]. In general phage display screening, known as biopanning, it is difficult to control the binding site of the selected phages, because the selection is performed against whole target proteins. To develop functional peptide ligands, a screening method yielding peptide ligands recognizing a specific structure of the target protein is required.

Several approaches for selecting ligands recognizing specific regions of the target protein have been reported. In some methods, an agonist corresponding to target antagonists [17], an unmodified state of target molecule [18], a different dimer state of the target multifunctional enzyme [19], and a target catalytic antibody inactivated with an irreversible inhibitor [20] have been used as target proteins for negative selection. Some other methods involving competitive selection using proteins from the same family [21], positive selection by using target proteins from different strains [22], elution with known ligands [23], or selection of partial peptides as target molecules [24] have also been reported. All these methods are only applicable to specific target proteins and not to proteins in general. Although a computational approach is an attractive option for determining a ligand-binding site [25,26], calculation of the binding ability is limited to short peptides.

**Figure 1.**
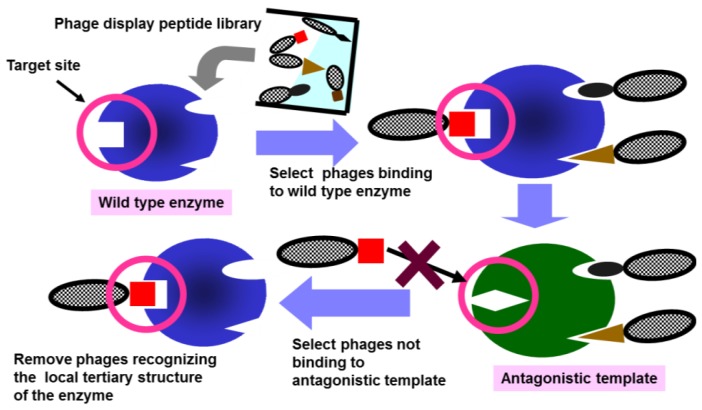
Schematic representation of the screening procedure for the peptide ligands involving mutant enzymes with disrupted local tertiary structures as antagonistic templates.

In this study, we propose a novel biopanning method to identify peptide ligands recognizing specific local structures of target proteins (Figure 1). In this method, mutant proteins are used as antagonistic templates for negative selection. In principle, the antagonistic templates harbor mutation(s) intended to disrupt local tertiary structure but not the scaffold structure of the target protein. To verify the usefulness of the method, water-soluble pyrroloquinoline quinone (PQQ) dependent glucose dehydrogenase (GDH-B) was used as a model protein. GDH-B is a representative β-propeller protein [27–29], which is composed of a simple building unit, having a highly symmetrical structure with 4–10 repeats of a four-stranded antiparallel β-sheet motif [30,31]. The propeller structure is a stable scaffold with the top, bottom, side faces, and the central channel capable of forming protein–protein interactions. Even substituting an entire four-stranded antiparallel β-sheet motif in PQQGDH with one from sialidase produced a chimeric PQQGDH showing similar structure and GDH activity [32]. The plasticity behind the rich functional diversity of β-propeller proteins is provided by the loops on the top and bottom faces of the propeller structure.

We [33], and other research groups [34,35], have been engaged in constructing of variety of mutant GDH-Bs in order to develop an ideal enzyme for glucose monitoring. For example, we have reported the Asn452Thr GDH-B mutant, showing approximately 50% lower activity for lactose and maltose than the wild-type GDH-B [36]. The Asn452 residue of GDH-B is located in loop 6BC (amino acids Thr449 to Gly468) and interacts with Tyr367 located in loop 4D5A (amino acids Tyr349 to Ser377) [28], suggesting that mutations in these loops resulted in local changes in tertiary structure in these loop regions. These studies demonstrated that the mutations within the loop regions did not significantly influence the overall structure, suggesting that the scaffold β-propeller structure can tolerate such mutations. We, therefore, selected the 4D5A and 6BC loop regions of GDH-B as target regions for the screening of peptide ligands and constructed two different antagonistic templates in which local tertiary structure had been disrupted in these loop regions. By using the mutants and wild-type GDH-B, we performed selection of peptide ligands that recognize the specific local tertiary structure within the loop regions of the wild-type enzyme.

## 2. Results and Discussion

### 2.1. Construction of Antagonistic Templates

**Figure 2.**
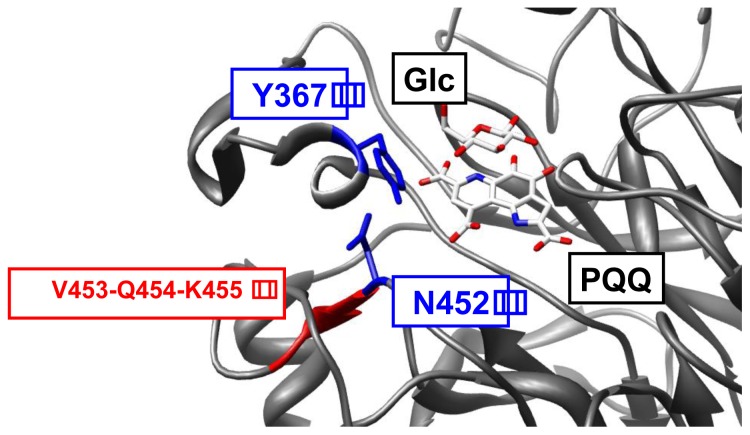
Structure of GDH-B around the target site with PQQ and β-d-glucose (PDB ID: 1CQ1). Blue boxes show the target position of HI, and the red box shows the target position of DEE. The image was produced with the program UCSF Chimera [37].

Two different GDH-B mutants, V453D-Q454E-K455E (DEE) and Y367H-N452I (HI), were constructed as antagonistic templates (Figure 2). The introduction of three consecutive negatively charged amino acids is expected to cause disruption of local tertiary structure in the loop 6BC of DEE. HI is a GDH-B mutant bearing one substitution in each of the two loops, 4D5A and 6BC. As Y367 and N452 form a hydrogen bond interaction, the local tertiary structure in HI is also expected to be altered.

**Figure 3.**
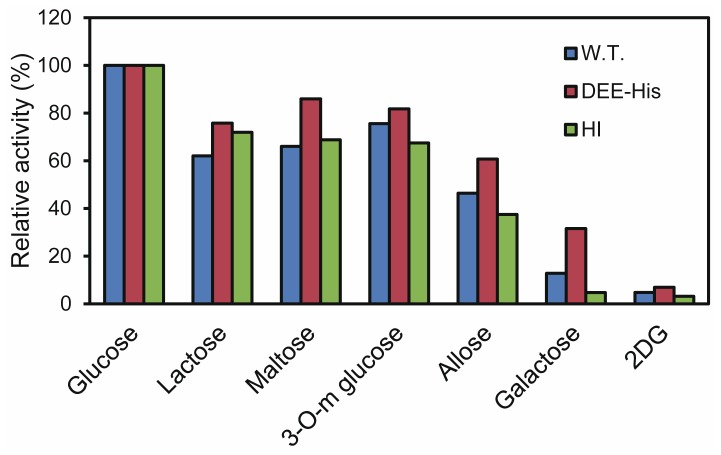
Substrate specificity of wild-type GDH-B (W.T.), DEE-His, and HI against glucose, lactose, maltose, 3-*O*-methylglucose (3-*O*-m glucose), allose, galactose, or 2-deoxyglucose (2DG). Each substrate concentration was 20 mM. The enzymatic activity of each enzyme against glucose was set as 100%.

Both, GDH-B mutants, and the wild type, were recombinantly produced in *Escherichia coli*. Wild-type GDH-B and HI were expressed in active soluble form. As DEE was expressed in an insoluble form as inclusion bodies, a 6-His tag was fused to the *C*-terminal (DEE-His) and active enzyme prepared by *in vitro* refolding. Investigation of their kinetic parameters shows an increased *K*_m_ value for DEE-His and decreased *V*_max_ values for both mutants, compared to the wild-type enzyme (Table 1). The substrate specificity of HI is similar to that of the wild-type enzyme, while DEE-His showed higher relative activity toward galactose (Figure 3). That these mutations resulted in altered catalytic efficiency indicates that both HI and DEE-His likely harbor different local tertiary structures around the active site from wild type.

HI was expressed in soluble form and could be purified by the same protocol (cation exchange chromatography) as wild-type GDH-B, indicating that its β-propeller scaffold structure was not significantly influenced by the two amino acid substitutions. As *E. coli* is not able to synthesize PQQ, the PQQGDH recombinantly expressed in *E. coli* were produced in apo-form and incubated with PQQ prior to the enzyme assay. The binding of PQQ requires multivalent interactions, including the binding of one molecule of Ca^2+^ in the active site, and the formation of β-propeller scaffold structure is essential [29]. The fact that the refolded DEE-His showed GDH activity with PQQ indicates that it retained the characteristic β-propeller scaffold structure of PQQGDH. The inability of DEE-His to fold properly in vivo may be due to inadequate flexibility of three consecutive mutations in the loop 6BC region. These results indicate that both HI and DEE-His likely have different local tertiary structures around the active site from wild type, while retaining the characteristic β-propeller scaffold structure of PQQGDH. We, therefore, concluded that these mutants could be suitable antagonistic templates for screening peptide ligands by phage display.

### 2.2. Antagonistic Template-Based Biopanning

A 12-mer random phage display peptide library was used for peptide selection. A phage titer greater than 4 × 10^11^ pfu was used in all rounds (Table S1). In the first and second rounds, GB-His (a GDH-B fused with 6-His tag to the *C*-terminal) was used as target protein to enrich the phage library that bound to GB-His. Selection pressure was maintained low by immobilizing a relatively large amount of GB-His (78 μg for the first round and 29 μg for the second round). Amino acid sequence analysis of the single phage clones after the second round of selection did not show significant convergence (Table S2), suggesting that the selected phage clones bound to different regions of GB-His, including the 4D5A and 6BC loops.

To enrich the phage library with peptides that recognize the 4D5A or 6BC loop regions, negative selection was performed in the third round using the antagonistic templates DEE-His and HI. The phages eluted in the second round were added at a similar titer (2 × 10^11^ pfu) to each antagonistic template, which were immobilized separately on different supports. The phages that did not bind to each antagonistic template were recovered and the amino acid sequences of the single phage clones were determined (Table S3). Considerable similarities were observed in the amino acid sequences. First, sequences bearing three or four sequential His residues and hydrophilic residues appeared with a high frequency. Second, sequences bearing one or two acidic residues and hydrophilic residues were identified.

**Figure 4.**
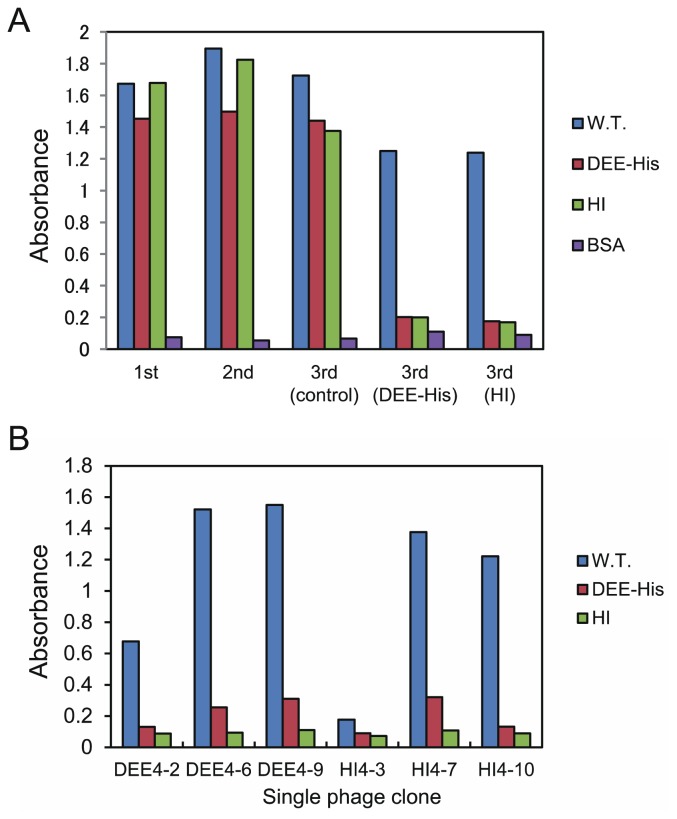
The evaluation of binding ability of screened phages by Phage ELISA. (**A**) Phage ELISA of the eluted phages from each round against GB-His (Blue bar), DEE-His (Red bar), HI (Green bar), and BSA for negative control (Purple bar); (**B**) Phage ELISA of the single phage clones from the fourth-round-eluted phages against GB-His (Blue bar), DEE-His (Red bar), and HI (Green bar).

Phage ELISA was performed to investigate whether the eluted phages from the third round specifically bound to GB-His. Second-round-eluted phages and third-round-eluted phages from the control-screening step (biopanning with only GB-His) showed binding to GB-His, DEE-His, and HI (Figure 4A). On the other hand, phages eluted from the third round (negative-screening round) bound to GB-His but did not bind to DEE-His or HI. These results reflect the clear effect of negative screening with antagonistic templates.

To obtain peptide ligands that bind to GB-His with greater affinity, we performed additional selection against GB-His with higher selection pressure (lower target quantity, shorter incubation time, and higher surfactant concentration) than that used for the first three rounds of screening. Similar to the sequences of the third-round-eluted phages, sequences bearing continuous His residues appeared with a high frequency and sequences bearing acidic residues were also noted (Table S4). Analysis by phage ELISA of the binding ability of the single phage clones DEE4-2, DEE4-6, DEE4-9, HI4-3, HI4-7, and HI4-10 demonstrated that most of the sequences, including those containing both sequential His residues and acidic residues showed specific binding to GB-His (Figure 4B).

### 2.3. Binding Assay for the Synthesized Peptides

We selected four different amino acid sequences from the third- and fourth-round-eluted phages and evaluated the binding ability of their synthesized peptides to wild-type GDH-B, DEE-His, and HI. The peptides HI3-2, DEE4–5 (same to DEE3-4), and DEE4–6 (same to DEE4–9) contain acidic residues in their *N*-terminal regions along with a relatively high number of hydrophilic residues. DEE4–10 (same as HI4–6) is a peptide bearing four sequential His residues and hydrophilic residues in its *N*-terminal region. DEE4–10 was the sequence that appeared in the fourth round in both the negative screening round of DEE-His selection and during HI selection. Using surface plasmon resonance (SPR) analysis, all peptides were confirmed to bind to GDH-B but not to DEE-His (Table 2). Although HI3-2 and DEE4–6 bound to HI, their dissociation constants were much higher than that for GDH-B. These results suggest that all peptide ligands recognize the 4D5A and 6BC loop regions of GDH-B.

### 2.4. Inhibition Assay for the Synthesized Peptides

**Figure 5.**
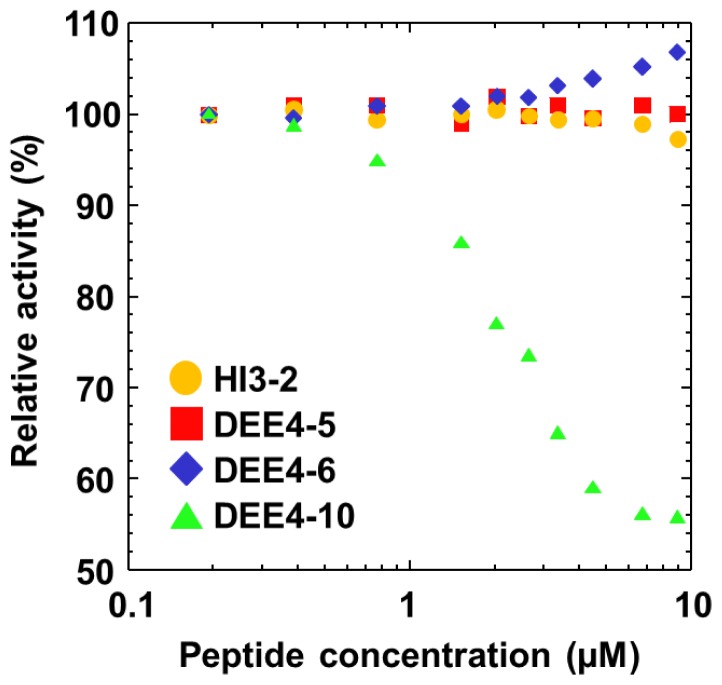
Residual activity of wild-type GDH-B in the presence of the synthetic peptide ligands. GDH-B activity was measured in the presence of HI3-2 (Orange circles), DEE4–5 (Red squares), DEE4–6 (Blue diamonds), or DEE4–10 (Green triangles) in 10 mM MOPS–NaOH (pH 7.0), 0.7 nM GDH-B, 3.3 μM CaCl_2_, 3.3 nM PQQ, and 50 mM d-glucose. The GDH-B activity in the absence of peptide ligands was defined as 100%.

As the 4D5A and 6BC loops are close to the active site of GDH-B, we investigated whether the selected peptide ligands inhibit wild-type GDH-B activity. Among the selected peptides, DEE4–10 showed non-competitive dominant mixed inhibition in a concentration-dependent manner with an inhibition constant (*K*_i_) of 7.4 μM (Figure 5). Although the 4D5A and 6BC loops are close to the active site, they do not form the active site. The non-competitive dominant mixed inhibition of DEE4–10 is therefore consistent with its specific binding to the 4D5A and 6BC loop regions. On the other hand, HI3-2, DEE4–5, and DEE4–6, which share a common sequence motif bearing acidic residues in their *N*-terminal region, did not inhibit GDH-B activity. DEE4–10 has a different sequence bearing sequential His residues, suggesting that although these peptide ligands recognize the 4D5A and 6BC loop region, the binding site of HI3-2, DEE4–5, and DEE4–6 differ slightly from that of DEE4–10.

In this study, we screened peptide ligands using two types of mutants as antagonistic template. One mutant disrupts loop–loop interactions and the other mutant has three consecutive negatively charged residues. These mutants were effective as antagonistic templates by breaking the local tertiary structure. Determining which amino acids to introduce at the target site is important in constructing an antagonistic template. As a target protein that undergoes a point mutation may retain the native tertiary structure, introducing multiple mutations may be more effective at disrupting the local tertiary structure. In this study, the stable β-propeller scaffold of our target GDH-B, would tolerate multiple mutations in the loop regions that are not related to the β-propeller scaffold formation. Most proteins have several loops that play a vital role in correctly positioning the catalytically important residues. Mutations in loop regions might affect their functions but not affect their overall structure. Antagonistic-template-based negative selection would be useful to obtain peptide ligands against loop regions. Screening for peptide ligands against other regions requires careful selection of the regions to ensure that mutations do not affect the overall structure. Furthermore, coexpression with chaperone proteins may help achieve proper folding for the expression of target mutant proteins in soluble form [38].

A molar excess of target antagonistic template, relative to the phage concentration (approximately 10:1 for DEE-His and 8:1 for HI), was used during the negative-screening round. This resulted in low selection pressure for antagonistic-template-binding phages and high selection pressure for recovering the phages that did not bind to the antagonistic templates. This regulation of selection pressure appears to have been effective in selecting phages that did not bind to the antagonistic template.

## 3. Experimental Section

### 3.1. Preparation of *C*-Terminal His-Tagged GDH-B (GB-His) and Antagonistic Templates

*E. coli* PP2418, in which the GDH-B structural gene was disrupted by insertion mutagenesis, was used as the host strain for the expression of GDH-B, GB-His, and the antagonistic templates DEE-His and HI [39]. All the GDH-B structural genes were inserted into the multi-cloning site of the expression vector pTrc99A. Mutan-Express Km (TaKaRa Bio Inc., Shiga, Japan) was used for the construction of the mutants. Wild-type PQQGDH-B and mutants were purified as previously described [40]. GB-His and DEE-His were purified using MagExtractor-His-tag- (TOYOBO, Osaka, Japan) according to manufacturer’s instructions, followed by Superdex 200 HR 10/30 size exclusion chromatography (GE Healthcare, Bioscience, Buckinghamshire, UK).

Inclusion bodies produced by *E. coli* PP2418/pTrc99A-GDH-B-DEE-His were washed by 1% Triton X-100 and denatured by 6 M Guanidine–HCl. The denatured enzyme was purified under denaturing conditions using MagExtractor-His-tag- and applied to a Bio-Select SEC 250-5 size exclusion chromatography column (BIO-RAD, Hercules, CA, USA). Purified enzyme sample was refolded by two-fold serial dilutions of Guanidine–HCl from 6 to 0.75 M by 10 mM MOPS–NaOH (pH 7.0) including 1 mM CaCl_2_. Enzyme solution at each Guanidine–HCl concentration was incubated 1 h at room temperature. Enzyme solution in 10 mM MOPS–NaOH including 0.75 M Guanidine–HCl was dialyzed in 10 mM MOPS–NaOH (pH 7.0), 1 mM CaCl_2_, and GDH-B-HI was purified with Resource S cation exchange column (GE Healthcare, Bioscience, Buckinghamshire, UK) and Superdex 200 HR 10/30 size exclusion chromatography column. Purification of the enzyme was confirmed by SDS-PAGE.

### 3.2. Screening Procedures

First round: The Ph.D.-12™ Phage Display Peptide Library Kit (New England Biolabs, Beverly, MA, USA) was used for biopanning. In the first round, 120 μg of GB-His in immobilization buffer (10 mM Tris–HCl (pH 8.0) containing 100 mM NaCl) was added to 120 μL of Ni-agarose magnetic beads of MagExtractor-His-tag- and rotated gently for 1 h at 4 °C. After immobilization of GB-His, the bead surface was blocked with blocking buffer (10 mM MOPS–NaOH buffer (pH 7.0), containing 1% BSA, 0.05% Tween 20, 1 mM CaCl_2_, and 1 μM PQQ) for 1 h at room temperature. The phage display peptide library (4 × 10^11^ pfu in blocking buffer) was incubated with Ni-agarose magnetic beads in microtubes to eliminate phages binding to the beads or the tubes. The library was subsequently added to the GB-His immobilized Ni-agarose magnetic beads and incubated for 6 h at 4 °C. Prior to incubation, the phages binding to Ni-agarose magnetic beads and support (microtube) were removed. This step was followed by five washes in blocking buffer and five washes in washing buffer (10 mM MOPS–NaOH buffer (pH 7.0), 0.05% Tween 20). The bound phages were eluted from the beads by 10-min incubation with 400 μL of elution buffer (0.1 M glycine–HCl (pH 2.2), 1 mg/mL BSA). The pH of the collected phage solution was neutralized by the addition of 60 μL of 1 M Tris–HCl (pH 9.5), and the phages were then amplified in *E. coli* ER2738 for the next round. Phage titration was performed by infecting *E. coli* ER2738 cells with varying dilutions of phages for 15 min at room temperature with no shaking. Two hundred microliters of this mixture was then mixed with top agar and immediately plated on l-broth agar plates containing IPTG (Isopropyl β-d-1-thiogalactopyranoside)/X-gal. The plates were incubated overnight at 37 °C, and visible blue plaques were counted and used for the next round.

Second round: the same procedure as the first round was followed, except that the first-round-eluted phages were incubated for 3 h at 4 °C with 48 μg of GB-His. The amount of GB-His not immobilized on the beads was quantified by measuring the quantity of protein in the supernatant, and it was confirmed that 78 μg (first round) and 29 μg (second round) of GB-His was immobilized.

Third round (negative-selection round): a similar procedure was used except that the antagonistic templates, DEE-His and HI, were used as targets. DEE-His was immobilized on 120 μL of Ni-agarose magnetic beads (33 μg of DEE-His was immobilized), and 100 μL of 20 μg/mL HI was immobilized in a well of a 96-well ELISA plate (Asahi Glass, Tokyo, Japan). Following the blocking step, 2 × 10^11^ pfu of second-round-eluted phages were added to each antagonistic template and incubated for 3 h at 4 °C. The phages that were obtained from positive selection and were not retained on these antagonistic templates were directly recovered and amplified for the fourth round of selection.

Fourth round: 2-μg wild-type GDH-B was immobilized in a well of a 96-well ELISA plate. After blocking, the third-round-eluted phages, both in the case of DEE-His selection and HI selection, were added separately to the wild-type GDH-B and incubated for 1 h at room temperature. This step was followed by washing five times with blocking buffer and five times with washing buffer. In this round, the concentration of Tween 20 in the blocking buffer and the washing buffer was increased to 0.1%. The bound phages were eluted from the plate by 10-min incubation with 100 μL of elution buffer (0.1 M glycine–HCl (pH 2.2), 1 mg/mL BSA). The pH of the collected phage solution was neutralized by 15 μL of 1 M Tris–HCl (pH 9.5), and the phages were amplified in *E. coli* ER2738 for the binding assay.

Control panning: As a control experiment, ordinary biopanning was carried out for the wild-type GDH-B. In the first and second rounds, GB-His was immobilized onto Ni-agarose magnetic beads, and in the third round, wild type GDH-B was immobilized into a well of a 96-well ELISA plate.

### 3.3. Phage ELISA

One hundred microliters of 10 μg/mL wild-type GDH-B, antagonistic templates (DEE-His and HI), and BSA (negative control) in 10 mM MOPS–NaOH buffer (pH 7.0) were coated into each well of a 96-well ELISA plate. The wells were blocked with blocking buffer. Variable amounts of phages (>2 × 10^9^ pfu per well) were added and incubated for 90 min in blocking buffer at room temperature. After four washes with washing buffer, bound phages were detected using 20 ng anti-M13 horseradish peroxidase-conjugated monoclonal antibodies (mAbs) (New England Biolabs, Beverly, MA, USA). The binding of labeled mAbs into the well was measured by the addition of 0.2 mg/mL 2,2′-azinobis(3-ethylbenzothiazoline-6-sulfonic acid) ammonium salt (Sigma-Aldrich, St. Louis, MO, USA), a peroxidase substrate, in 50 mM citrate buffer (pH 4.0) with 0.05% H_2_O_2_. Absorbance was read at 405 nm.

### 3.4. Assay for the Binding of the Synthesized Peptide to Wild-Type GDH-B and to the Antagonistic Templates

The synthesized peptides HI3-2, DEE4–5, DEE4–6, and DEE4–10 were purchased from Sigma Aldrich Japan, Tokyo, Japan. The purity was greater than 95%. SPR analysis was performed with a BIAcore X system (GE Healthcare, Bioscience, Buckinghamshire, UK). The peptide (200 μg/mL in 10 mM acetate (pH 4.0), 400 mM NaCl) was immobilized onto the surface of a CM5 chip by injecting 100 μL at a flow rate of 5 μL/min, using the standard amine coupling procedure. The RU value corresponding to immobilization was 320–2130 RU for each peptide. As analytes, five reagent concentrations (5 nM to 90 μM) of wild-type GDH-B, the antagonistic templates DEE-His or HI in 10 mM MOPS–NaOH buffer (pH 7.0) were injected at a flow rate of 10 μL/min until the SPR signal reached a plateau. The *K*_d_ value was determined by plotting the RU at the plateau of the binding curve versus the analyte’s concentration. In all Scatchard plots, the coefficient of determination was greater than 0.9. When no significant response (less than 20 RU) was observed for any analyte concentration, it was judged as not binding.

### 3.5. Inhibition Assay for the Synthesized Peptide and Wild-Type GDH-B

GDH-B activity was measured using 0.06 mM 2,6-dichlorophenolindophenol (DCIP) and 0.6 mM phenazine methosulfate (PMS) following 30 min pre-incubation in 10 mM MOPS–NaOH buffer (pH 7.0) containing 1 μM PQQ and 1 mM CaCl_2_ at room temperature (25 °C). The enzyme activity was determined by measuring the decrease in absorbance of DCIP at 600 nm [35]. For the inhibition assay, the residual activity of 0.7 nM GDH-B for 50 mM d-glucose was measured following 1 h incubation with various concentrations of each peptide. GDH-B activity in the absence of peptide was defined as 100%.

## 4. Conclusions

We have developed a novel screening method using antagonistic templates with multiple point mutations in the target region for screening of peptide ligands recognizing local tertiary structure of a target protein. We obtained several peptide ligands that bind to wild-type GDH-B but not to the antagonistic templates. To screen for site-directed peptide ligands using antagonistic template, the nature of the mutations introduced to the target site are important. In this study we constructed two types of effective antagonistic templates whose local tertiary structure would be disrupted. Site-directed ligands have a wide range of useful applications, including as inhibitors or specific molecular recognition elements. Our novel strategy can be applied to proteins whose overall scaffold structures are not affected by the introduced mutations. In addition to peptide ligands, our strategy would also enable the screening of aptamers and antibody single-chain variable fragments. We believe this methodology will be beneficial for obtaining useful ligands for many different applications.

## Supplementary Information



## Figures and Tables

**Table 1.** Kinetic parameters of wild-type glucose dehydrogenase (GDH-B), His-tagged GDH-B (GB-His), Y367H-N452I (HI), and His-tagged V453D-Q454E-K455E (DEE-His).

Name	*K*_m_ (mM)	*V*_max_ (U/mg protein)
GDH-B	25	5200
GB-His *	24	3950
HI	18	170
DEE-His	91	28

* GB-His is a GDH-B fused with 6-His tag to the *C*-terminal.

**Table 2.** Dissociation constant of selected 12-mer peptide ligands against GDH-B, HI, and DEE-His. (N.B. means not bound).

Name	Sequence	*K*_d_ (μM)		

		GDH-B	DEE-His	HI
DEE4–5	LGDSSNSQVSLN	13	N.B.	N.B.
DEE4–6	SDLSPIQSLSAI	2.5	N.B.	230
DEE4–10	NSTHHHHFATIW	0.7	N.B.	N.B.
HI3-2	ELITNSETTQWF	2.5	N.B.	88
