# Supplementary material for: Screening of Peptide Ligands for Pyrroloquinoline Quinone Glucose Dehydrogenase Using Antagonistic Template-Based Biopanning
Source: Int J Mol Sci. 2013 Nov 25;14(12):23244–56. doi: 10.3390/ijms141223244 (PMC3876041; doi:10.3390/ijms141223244)
Supplement: Supplementary file 1 [file ijms-14-23244-s001.pdf]

Supplementary Information

Table S1. Phage titer of each round.

Round	Input phage (pfu)	Eluted phage (pfu)	Amplified phage (pfu)
1st round	4×10^{11}	4×10^{4}	3×10^{18}
2nd round	3×10^{15}	5×10^{6}	5×10^{22}
3rd round (DEE-His)	2×10^{11}	7×10^{3}	5×10^{16}
3rd round (HI)	2×10^{11}	1×10^{5}	2×10^{18}
4th round (DEE-His)	5×10^{14}	2×10^{7}	3×10^{22}
4th round (HI)	2×10^{16}	1×10^{8}	6×10^{23}

Table S2. Amino acid sequences of the eluted phages from the second selection round.

Clone No.	Amino acid sequence
2-1	LDTHASHACSTG
2-2	GPIPGLLATVAV
2-3	SVVTSHQRYGTS
2-4	SNSPTFVCHRMV
2-5	GPIPGLLATVAV
2-6	VQFPIEAMFWST
2-7	MATQPKLVGSPY

Table S3. Amino acid sequences of the eluted phages from the third selection round.

Clone name	Antagonistic template	Amino acid sequence
DEE 3-1	DEE-His	WPYNHHHRTPSP
DEE 3-2	DEE-His	LPHSAVMAQLTY
DEE 3-3	DEE-His	YSHHHMHTPHTR
DEE 3-4	DEE-His	LGDSSNSQVSLN
DEE 3-5	DEE-His	WMNGPVSIRTWS
DEE 3-6	DEE-His	TLHAHQHHQPST
DEE 3-7	DEE-His	STSHHHHPSAPS
HI 3-1	HI	TMQPGQNSHPIL
HI 3-2	HI	ELITNSETTQWF
HI 3-3	HI	APLSQHHHHLRP
HI 3-4	HI	TSHIHTTPHSHH
HI 3-5	HI	YNHHGHHLDKHR
HI 3-6	HI	YHPANHSFQHHF
HI 3-7	HI	MHDLTAALSLPP

Table S4. Amino acid sequences of the eluted phages from the fourth selection round.

Clone name	Antagonistic template used at 3rd round	Amino acid sequence
DEE 4-1	DEE-His	APYAHHHHPVTP
DEE 4-2	DEE-His	EELWHHHPPSHH
DEE 4-3	DEE-His	TSLHQHHPTAAF
DEE 4-4	DEE-His	QPHKYPHSHHGP
DEE 4-5	DEE-His	LGDSSNSQVSLN
DEE 4-6	DEE-His	SDLSPIQSLSAI
DEE 4-7	DEE-His	APLFTQTWGPWR
DEE 4-8	DEE-His	VAQHSHHHVTPS
DEE 4-9	DEE-His	SDLSPIQSLSAI
DEE 4-10	DEE-His	NSTHHHHFATIW
HI 4-1	HI	SPHSHHMSPSEY
HI 4-2	HI	QPHKQAVSFAFA
HI 4-3	HI	VHTHHLGHQPVR
HI 4-4	HI	QTSHYHHHRAHT
HI 4-5	HI	ANPLHHHHLWEL
HI 4-6	HI	NSTHHHHFATIW
HI 4-7	HI	LSPHHHHLDGHI
HI 4-8	HI	SPHSHHMSPSEY
HI 4-9	HI	LSPHHHHLDGHI
HI 4-10	HI	TPTFPYWYGSLT

© 2013 by the authors; licensee MDPI, Basel, Switzerland. This article is an open access article distributed under the terms and conditions of the Creative Commons Attribution license (http://creativecommons.org/licenses/by/3.0/).
